# Multi-Channel Bioimpedance System for Detecting Vascular Tone in Human Limbs: An Approach

**DOI:** 10.3390/s22010138

**Published:** 2021-12-26

**Authors:** Ahmad Hammoud, Alexey Tikhomirov, Galina Myasishcheva, Zein Shaheen, Alexander Volkov, Andrey Briko, Sergey Shchukin

**Affiliations:** 1 Department of Medical and Technical Information Technology, Bauman Moscow State Technical University, 105005 Moscow, Russia; tikhomirov.an@bmstu.ru (A.T.); myasishcheva@bmstu.ru (G.M.); briko@bmstu.ru (A.B.); schookin@bmstu.ru (S.S.); 2 Department of Informatics and Applied Mathematics, ITMO University, 197101 St. Petersburg, Russia; shaheen@itmo.ru; 3 Scientific and Educational Medical-Technological Center, Bauman Moscow State Technical University, 105005 Moscow, Russia; akv3011@bmstu.ru

**Keywords:** vascular tone, arterial stiffness, hemodynamic, blood flow, bioimpedance, graphical interface, signal processing

## Abstract

Vascular tone plays a vital role in regulating blood pressure and coronary circulation, and it determines the peripheral vascular resistance. Vascular tone is dually regulated by the perivascular nerves and the cells in the inside lining of blood vessels (endothelial cells). Only a few methods for measuring vascular tone are available. Because of this, determining vascular tone in different arteries of the human body and monitoring tone changes is a vital challenge. This work presents an approach for determining vascular tone in human extremities based on multi-channel bioimpedance measurements. Detailed steps for processing the bioimpedance signals and extracting the main parameters from them have been presented. A graphical interface has been designed and implemented to display the vascular tone type in all channels with the phase of breathing during each cardiac cycle. This study is a key step towards understanding the way vascular tone changes in the extremities and how the nervous system regulates these changes. Future studies based on records of healthy and diseased people will contribute to increasing the possibility of early diagnosis of cardiovascular diseases.

## 1. Introduction

The industrialized world is witnessing some common and costly diseases such as those related to cardiovascular issues.Cases of total cardiovascular diseases (CVD) nearly doubled from 271 million in 1990 to 523 million in 2019, and the number of CVD deaths steadily increased from 12.1 million in 1990 to 18.6 million in 2019 [1,2,3]. In this regard, it is important to mention that changes in vessel tone are important in regulating blood flow in arteries and blood pressure in general. As a result, these changes have direct effects in regard to hypertension and coronary artery disease. Blood vessels constrict and expand according to different conditions. The ratio between diameter of a blood vessel when it is constricted and when it is expanded to its maximum value indicates the vessel’s tone (vascular tone). In basic conditions, most blood vessels contain flexible muscles (smooth muscles) that are capable of constricting to a certain degree. The constriction of these flexible muscles determines the blood vessel tone. Usually, both endothelium and vascular smooth muscle (VSM) affect the vascular tone [4,5].

Factors affecting vascular tone are classified into two types: external factors and internal factors. External factors are originally from sources outside of the blood vessel. Internal factors are from the vessel itself. The external factors basically regulate blood pressure inside arteries by modifying the systemic resistance of blood vessels. Internal factors participate in regulating local blood flow inside a human organ [5].

Vascular tone of resistance arteries and arterioles determine peripheral vascular resistance, contributing to the regulation of blood pressure and blood flow to and within the body’s tissues and organs. In fact, there are two factors which determine vascular tone of the resistance arteries and arterioles. The first factor is blood pressure within the vessels. The other factor is the balance between vasoconstrictor and vasodilator signals which run into each other in these vessels. The wall of the resistant arteries and arteriole is made up of vascular smooth muscle cells (SMCs). These cells work as primary effectors in the minute-to-minute active regulation of vascular resistance. This operation is done via modulating the steady-state contraction of either these cells or the vascular tone. Myogenic tone is produced by blood pressure which stretches the SMCs activating signaling pathways. This tone distinguishes the resistant arteries and arterioles. Myogenic tone is the baseline smooth muscle cell contraction. When this contraction occurs, signals which express vessels expansion and constriction coming from different sources act. Sources of these signals are neurotransmitters, hormones, endothelium derived substances, local metabolites and ions [6,7,8,9,10,11,12].

The plasma membrane and endoplasmic reticulum of SMCs in blood vessels contain ion channels. These channels play the most important role in regulating intracellular Ca2+ concentration. Moreover, these channels primarily determine both smooth muscle cell contractile activity and vascular tone [13].

Some afferent nerves have an efferent (motor) function, and axon reflex control of vascular tone by these “sensory-motor” nerves is more widespread than once thought. Endothelial cells arrange both vessel expansion (vasodilatation) and constriction (vasoconstriction). These endothelial cells are able to store and emit substances which are active in the vessels. These substances are called vasoactive substances. Acetylcholine (vasodilator) and endothelin (vasoconstrictor) are examples of these substances. Endothelial vasoactive substances may be more significant when blood vessels respond to local changes [14].

There are many factors which affect vascular tone regulation. Some of these factors include aging and other conditions such as hypertension, trauma and surgery. Vascular tone has been studied in case of disease, after denervation, or after mechanical injury. In all of the cases, these studies showed possible alimental interactions between the perivascular nerves and the endothelial cells. Such alimental interactions may be important for growing and developing both of the control systems. This case is particularly clear in the microvasculature where the neural-endothelial separation is small [15].

### 1.1. Vascular Tone Measurement

Saito et al. developed an ultrasonic probe [16]. The probe can measure blood pressure and blood vessel diameter changes at the same position in the radial artery. Zlepko et al. evaluated vessels’ tone and characteristics of the local blood flow using a photoplethysmographic device. The primary investigation areas were fingers, toes, the prebrachial joint, and the middle third part of the tibia [17]. Tusman et al. attempted to detect changes in systolic arterial blood pressure (SAP) and vascular tone using a classification based on the contour of the photoplethysmography signal (PPGc). The obtained results showed that changes in arterial pressure and vascular tone were closely related to the proposed classification based on PPG waveforms [18].

Kellogg and Dean had focused on the neural and the local mechanisms that influence cutaneous vasodilatation and vasoconstriction when responding to heat and cold pressure in humans. There is no knowledge about non-neural mechanisms that mediate the prolonged response to local cooling. The involved mechanisms that may be included are changes of endothelial function, blood viscosity, receptor affinity, and/or coriaceous vascular smooth muscle function. None of these mechanisms have yet to be investigated in humans [19].

Wang et al. performed a non-invasive estimation of the vascular tone changes. The estimation was performed by observing variations in response of a photoplethysmogram and a novel piezoelectric cardiovascular sensor. The results of tracking vascular resistance showed strong correlation with invasive systems that measure vascular resistance in swine subjects, altering muscle vascular tone: endothelial, neurogenic and myogenic, spatially localized in the microvasculature. They create fluctuations in blood flow in known frequency ranges (0–1.6 Hz). The software used for registering and processing the laser doppler-flowmetry-gram allows diagnosing activity of a certain regulatory mechanism. The aser doppler-flow-metry method makes it possible to assess components of microvessels’ tone based on magnitudes of microcirculation oscillations amplitudes [20,21].

Impedance plethysmography (rheography) is a biophysical way for studying blood circulation non-invasively. This method is based on regista ering and medically analyzing changes in the variable component of electrical impedance to high-frequency current. Pulse fluctuations in blood flow under influence of an intra-arterial pressure gradient cause a change in the complex electrical resistance. This happens due to the arrival of a pressure wave generated by cardiac contractions into the studied area [22]. The state of the cardiovascular system is usually studied based on rheography. It is able to identify pathologies associated with both violations of the tone of individual vessels and systemic hemodynamics in general [23,24].

Previous studies of vascular tone focused on local measurement and specific arteries. However, the studies were not for several arteries or segments at the same time. Therefore, previous studies give answers about the change in the type of vascular tone in the studied artery, but they cannot give information about the changes in the vascular tone in the different arteries of the body at the same time. Bioimpedance technology using a multi-channel device when used to measure vascular tone will allow comprehensive monitoring of vascular tone changes associated with blood pressure in different arteries of the human body. The aim of this work is to present a methodology that allows for the monitoring of vascular tone changes in different parts of the human body (peripheral arterioles) simultaneously.

### 1.2. Problem Statement

In this work, there are three main tasks to be accomplished. The main task is to identify the vascular tone in all parts of human body for each cardiac cycle. The second task is to determine whether this cycle is within inspiration or exhalation in order to know the relationship of the change in the type of vascular tone with the change in the breathing phase. The third task is to classify the main types of signals that can be obtained in human limbs over a long period of time, and then to create a graphical interface to demonstrate the vascular tone type during the whole record.

### 1.3. Impedance Plethysmography for Detecting Vascular Tone

In the bioimpedance signal in extremities, several points can be identified. These points are often found in each cycle and they are as follows. S is the beginning of systolic wave, C is the first systolic wave peak, I is the incisura, and D is the diastolic wave peak; In the ideal case, these points reflect the change in blood volume for specific arteries, veins, and blood capillaries for a specific part of the body and in a certain health condition. This means expansion and contraction of these points during the signal recording is compatible only with the pressure and volume of the blood flow. Blood flow in veins and capillaries is considered steady and not pulsating [25,26]. Therefore, pulsational changes, recorded by the change of bioimpedance, are mainly due to changes in blood flow in the arteries. In fact, change in blood flow is related to change in pressure, which in turn leads to a change in the diameter of the artery. Therefore, vascular tone changes can be calculated based on bioimpedance signals.

**Figure 1:**
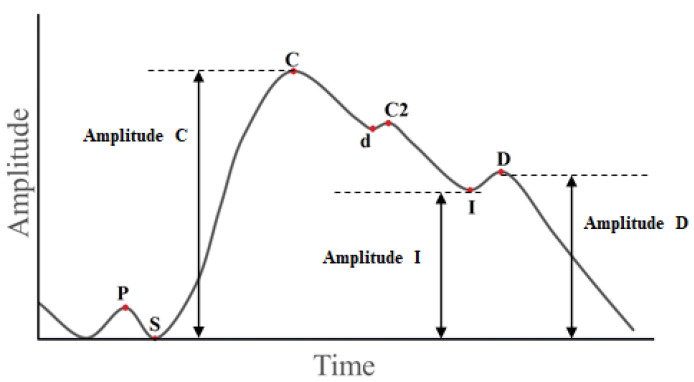
The ideal bioimpedance signal in the extremities, **P** is the presystolic wave peak, **S** is the beginning of systolic wave, **C** is the first systolic wave peak, **C2** is the second systolic wave peak, **d** is the dicrotic wave peak, **I** is the incisura, and **D** is the diastolic wave peak.

In this work, a system for monitoring changes in vascular tone has been proposed. A monitoring process has been undertaken for all segments of the body’s limbs simultaneously. The monitoring operation of the limbs has been linked to breathing and ECG. The purpose is forming a system for displaying changes in vascular tone simultaneously based on multi-channel bioimpedance. Using this system, the changes can be monitored. Moreover, this system can be used to better understand the nature of the change in blood vessel tone. The proposed system can be prepared to explain the mechanism according to which the nerve system behaves in various conditions. In these conditions, the nerve system must regulate vessel tone. Some examples of these conditions include cold, heat, exercise, stress, etc. Two parameters must be calculated in order to determine the type of vascular tone based on the bioimpedance signal. Each one of these two parameters depends on the systolic wave amplitude, the diastolic wave amplitude, and the incisura. Figure 1 shows the ideal signal in the extremities in which the systolic wave and the diastolic are clear, so that we can clearly define the amplitude of points C, I, and D with a noticeable difference in the their value to determine the vascular tone.

The first systolic wave arises as a result of the interaction of the pumped volume of blood into the arterial bed and the resistance of this part of the vascular system. The second systolic wave is formed as a result of reflection from the aortic bifurcation, therefore it does not occur or is extremely weakened on the rheovasogram of the extremities. The diastolic wave is the result of reflection from the peripheral part of the arterial tree, the smallest arteries and arterioles. As the elasticity of the arteries is lost, the formation of the diastolic wave is increasingly affected by additional reflection waves from more distant, proximally located sections of the arterial bed. Incisura between the systolic and diastolic waves is formed as a result of their addition and its depth depends on the formation and interaction of these waves.

It is required to determine the vascular tone type that expresses expansion of the blood vessel when a pressure wave passes. For this purpose, the dicrotic index (DKI), which reflects the level of tone of small vessels, and the diastolic index (DCI) which reflects the state of venous outflow, have been calculated using the following equations:

(1) DKI=AI/AC

(2) DCI=AD/AC

AC, AI, AD are amplitudes of the systolic wave peak, incisura and diastolic wave peak, respectively. Based on DKI-DCI phase plane, type of the tone can be determined. This tone has three main types: normotonic, hypotonic, and hypertonic [27,28]. However, in case the tone type is monitored for a relatively long period, it is necessary to identify the cycles in which the tone type is detected, and to identify whether these cycles are within inhalation or within exhalation. In addition to that, it is necessary to separate changes in the signal due to breathing. Respiration can affect both the vascular tone itself, and the bioimpedance signal’s baseline instability. If the amplitude is defined at any point in the signal without removing the breathing effect (base line drift), the amplitude value of any point will not be accurate, and therefore the determination of the vascular tone type will not be accurate. These changes in signal by breathing do not express changes in blood vessels diameters in response to the change in the current pressure.

The point S expresses beginning of arterial blood wave arrival to the studied segment. By this point, the vessels begin to expand and the blood stream enters the small arteries and capillaries in the segment. At this time, processes of gas exchange and energy transfer to the tissues in the studied segment begin. In fact, the flow is at its beginning in arterial blood vessels section. In the veins and their branches, there is blood stream in the opposite direction. This stream is small in relation to the small ratio of venous pressure versus to arterial pressure. This point can be relied on to normalize baseline of the bioimpedance signal and to approximate the signal to the ideal state.

## 2. Materials and Methods

### 2.1. Subjects

The study included 14 volunteers (seven male and seven female) which, according to the Ethics Committee, is the minimum number of studied subjects to make a classification of bioimpedance signals types in human limbs. The volunteers, whose ages are in the range 25–33 years are non-smokers. They don’t have diseases related to respiratory system or cardiovascular diseases.The body mass index (BMI) range of the subjects was 18.5–35. The blood pressure and heart rate of all volunteers were measured before the start of recording the signals and they were within the normal limits.

The experiments have been conducted under supervision of the Medical and Educational Center of Bauman Moscow State Technical University.The study followed the World Medical Association’s Declaration of Helsinki on Ethical Principles for Medical Research Involving Humans Subjects. All patients provided written consent before they participated in the study.

### 2.2. Experimental Procedure

During the experiments, the volunteers were lying down fully relaxed exactly imitating the patient’s position under observation. Recording the bioimpedance signals had begun after 10 minutes of complete relaxation. The signals were recorded for 7–10 minutes in free breathing without interruptions.

### 2.3. Equipment

The equipment used in this research is the multi-channel electrical impedance research system REO-32. This system has the following characteristics: 30 precordial electrical impedance channels, one transthoracic bioimpedance channel, one ECG channel, channel sampling rate is 500 HZ, bioimpedance measurement method is tetropolar, probe current amplitude is 1 mA, probing frequency is 100 kHz, pulse impedance measurement range is (−2∼+2) Ohm, and bandwidth of the bioimpedance channel is (0.01∼117) Hz. Each electrode has been connected to contact point of the limb symmetrically from two sides. Thus, eight electrodes per channel are required. CERACARTA Top Trace ECG Electrodes 50 mm with Ag/AgCl sensors have been used in the experiments.

**Figure 2:**
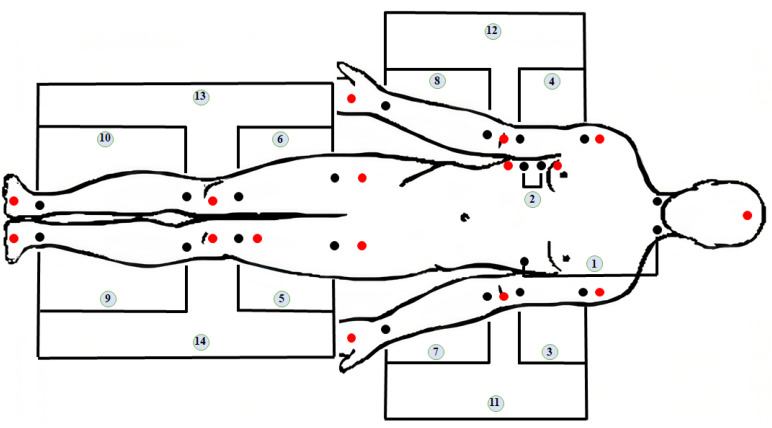
Used channels and placement of electrodes (1-Transthoracic, 2-Thoracic, 3-upper part of left arm (LAU), 4-upper part of right arm (RAU), 5-left thigh (LLU), 6-right thigh (RLU), 7-lower part of left arm (LAD), 8-lower part of right arm (RAD), 9-lower part of the left leg (LLD), 10-lower part of the right leg (RLD), 11-left arm (LA), 12-right arm (RA), 13-right leg (RL), 14-left leg (LL)).

In addition to the ECG signal, the signals have been recorded from 14 major segments of the body. Measurement points for these signals are shown in Figure 2. In Table 1, we present main information about the recorded signals, the index of the signal in connection with Figure 2, the segment of the body from which we recorded the signal and the main arteries in this segment.

### 2.4. Signal Processing

First of all, signals must be smoothed in one way to ensure that amplitude losses due to smoothing will be the same across all signals. Many methods can be used for smoothing the bioimpedance signal. The main methods for this purpose are fast Fourier transforms (FFT), Binomial smoothing, Locally Weighted Polynomial Regression Method (LOESS), and Savitsky-Golay. In this research, fast Fourier transforms have been used in addition to the use of Binomial smoothing in special cases. These cases were explained in detail in [29].

Processing ECG and detecting R peaks were done by library Heartpy based on [30]. Interpolation for S or C points can be done by different orders. For biomedical signals, it was recommended to use cubic interpolation in many works such as [30,31].

**Figure 3:**
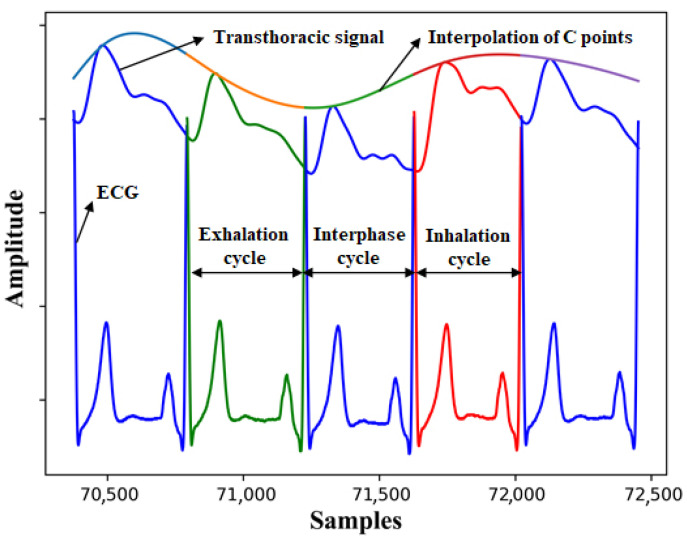
Dividing signals intervals within inhalation, exhalation, or in interphase based on ECG and trancthoracic signals.

**Figure 4:**
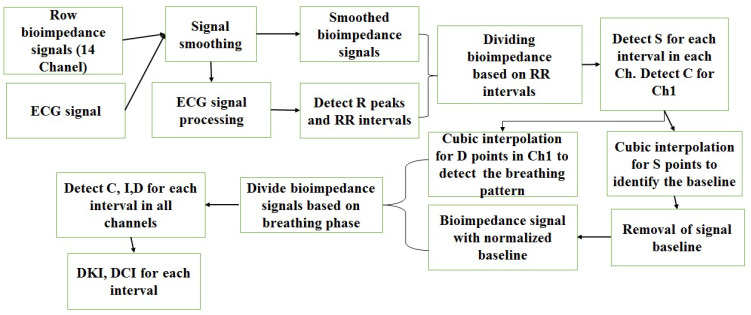
Basic signal processing stages.

As shown in Figure 3, dividing intervals within inhalation, exhalation, or in interphase can be done by the transthoracic signals and C points for each interval. After forming a line connecting the points C for each interval in Ch1, the intervals can be divided into inhale and exhale. Division is done based on height of the line towards the top. This upward direction represents inhalation and its slope downward represents exhalation. Between inhale and exhale, there are intervals. It is not possible to accurately determine whether these intervals belong to inhale or to exhale [30,32,33]. All processing stages have been implemented in Python 3. Figure 4 shows all the basic processing stages.

## 3. Results

In the study, several smoothing methods have been tested. These methods are Savitzky-Golay, LOESS, fast Fourier transforms (FFT), and Binomial smoothing. All of these methods achieved the desired goal with some differences in terms of displacement of signal peaks from the original signal when using large values for smoothing coefficients.

However, FFT ineffectiveness has been also observed in the presence of signals where the diastolic wave peak is very small. This peak can be lost due to excessive smoothing; the smoothing process was explained in our previous work [29].

**Figure 5:**
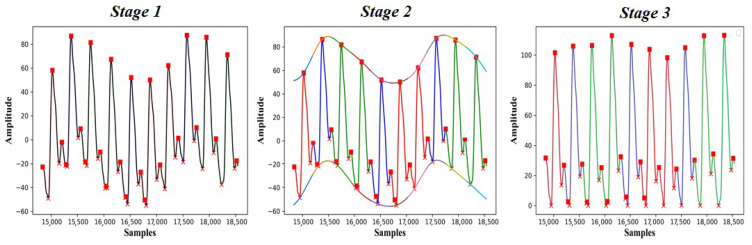
Main stages of removal of signal baseline.

The signal baseline has been removed in three main stages, Figure 5. It has been done depending on point S for each cycle, then it has been connected with a spline, after that it has been subtracted from the signal. It is not recommended to use peak C as the value of this point changes due to stroke volume variation (SVV). The breathing curve has been extracted based on the curve which connects the peaks C for each cycle of ch1. The cycles have been divided into inhalation, exhalation and interphase cycles based on the slope of the curve. After that, cycle type has been linked to all other channels.

Figure 5 shows the main stages of removal of the signal’s baseline. The green color represents exhalation, the red color represents inhalation, and the blue color represents the cycles between inhalation and exhalation.

**Figure 6:**
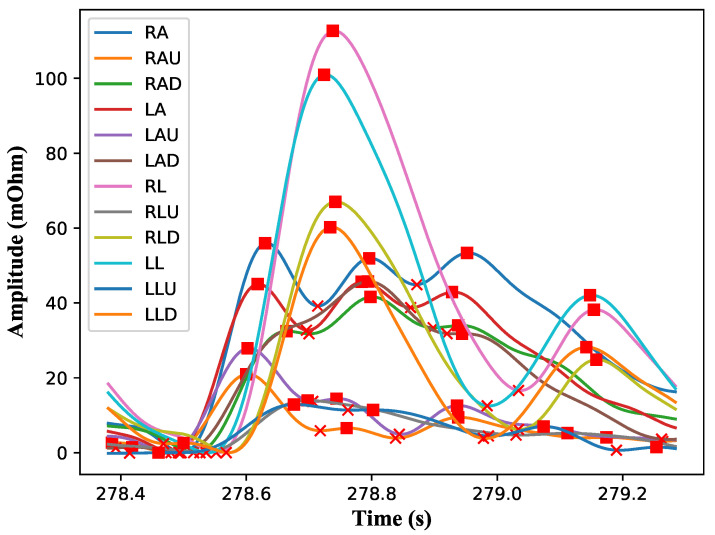
Signals in all channels after the completion of the processing process (Subject 1, Interval 300, Inhalation).

**Figure 7:**
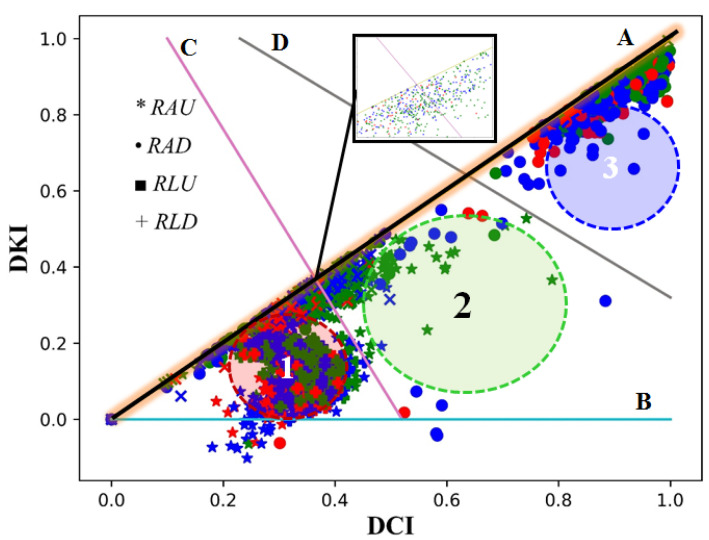
Main areas for vascular tone classifications: 1-Hypertonic, 2-Normotonic, 3-Hypotonic; red: inhalation, green: exhalation, and blue: interphase.

After smoothing the signal, removing the baseline and dividing the cycles into inhalation and exhalation, the type of vascular tone can be determined as shown in Figure 6. This can be done by calculating the coefficients DKI and DCI for each cycle. After that, the type can be determined based on the ratio between these coefficients as shown in Figure 7. A literature review shows that there are three main areas for classifying the tone type. Areas 1, 2, and 3 correspond to the hypertonic, normtonic and hypotonic tones, accordingly. The classification was done according to the Luzhnov and Shamkia classification [27,28].

**Figure 8:**
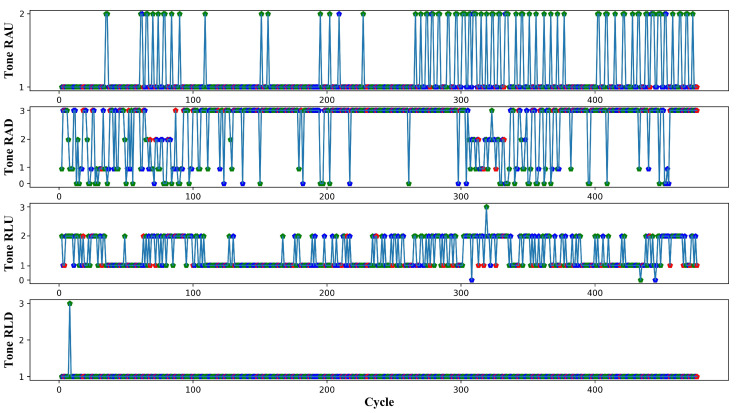
Vascular tone changes for each ECG cycle for subject 1 in channels RAU, RAD, RLU, and RLD; Vascular tone type: 1-Hypertonic, 2-Normotonic, and 3-hypotonic; red: inhalation, green: exhalation, and blue: interphase.

In order to track changes of vascular tone type with time, a chart has been created for this purpose. It determines the type of vascular tone for each cardiac cycle. Moreover, it determines whether a signal is within the inhalation phase or the exhalation phase (green: exhalation, red: inhalation, and blue: cycles between inhalation and exhalation) as shown in Figure 8.

**Figure 9:**
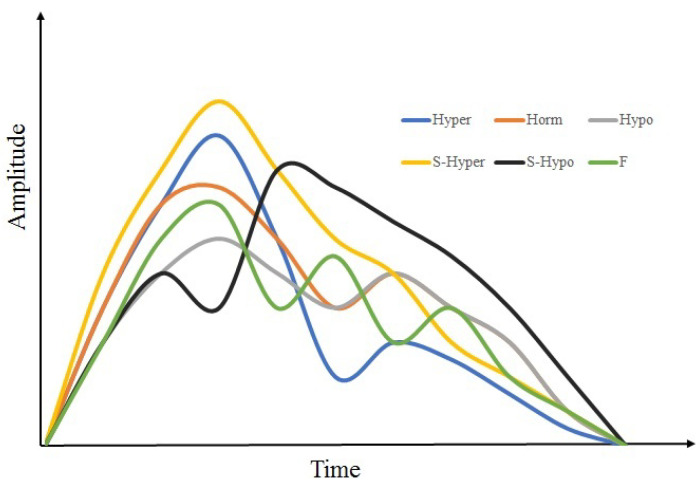
Basic classes of bioimpedance signals in human limbs.

In all the studied subjects, it has been found that there are six basic types of signals which can appear clear and stable during the recording period. Figure 9 presents basic classes of the signals that can be observed when recording bio-impedance signals in the human limbs. Three signals were previously classified as normo, hyper and hypo [28,34]. The S-hypo signal appeared only in the upper limbs of some subjects. It didn’t appear in the lower limbs of all subjects. The S-hyper signal (the black signal in Figure 9, when the diastolic wave was bigger than the systolic wave) appeared only in the lower limbs of some subjects, and didn’t not appear in the upper limbs of all subjects. Signal type F was clearly observed in female subjects where the third wave was clearly visible in recorded signals of some female volunteers.

**Figure 10:**
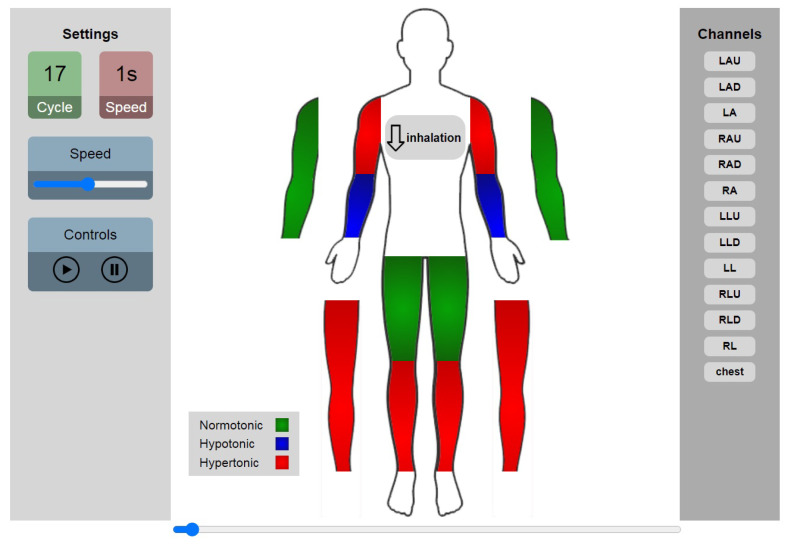
Vascular tone types and their distribution in the limbs for S1 cycle 17; red: hypertonic, blue: hypotonic, and green: normotonic.

In order to visualize changes of the vascular tone type in all segments, the graphical interface shown in Figure 10 has been created (supplemetary materials contain a video of tone changes). The type of the vascular tone has been represented by color of the segment for each cardiac cycle. The red color refers to the hypertonic type, the green color refers to the normtonic, and the blue color refers to the hypertonic. Segments without color during display, indicate the absence of the diastolic wave (S-hyper) from the signal or S-hypo type in this cycle. The breathing phase in the thoracic region has been also shown in Figure 10.

## 4. Discussion

It was clarified earlier that this study aims at presenting a practical method for observing changes in vascular tone in the extremities. Observation has been done from the stage of recording the signal until the stage of showing the type of vessel tone. This study is based on the bioimpedance signals in segments of the limbs, in the entire limbs, and related to the respiration phase. Based on the signals obtained during recordings, main types of the signals which may appear have been indicated as clear from Figure 9. A detailed explanation of signal processing stages has been given and the methods which can be used have been reviewed.

### 4.1. Method Performance

The followed method allows for the determination of the type of the vascular tone in the extremities. The challenges lie in finding one specific smoothing method suitable for all the signals that can be recorded. FFT shows good efficiency and can be adopted only in the presence of S-hyper type signals. Removal of the signal baseline has been done using an effective performance method. However, some errors may appear in some cases during long recording periods due to lack of signal stability, as when the patient moves, for example.

### 4.2. Vascular Tone Type

The following ideas can be discussed when determining the type of the vascular tone and based on the DKI and DCI values (see Figure 7). If a point formed by the values of DKI and DCi is located in areas 1, 2 or 3, the type of the vascular tone can be determined simply according to Luzhnov and Shamkina classifications. According to their classifications, area 1 (between line A and line C) represents hypertonic, area 2 (between lines C, A, and D) represents normatonic, and area 3 (between line D and line A) represents hypotonic. In case the point approaches line A in Figure 7 the reliability of the automated classification becomes low, since the points located in this area are considered unreliable as point I (incisura) is very close to the peak of the diastolic wave D. This case requires a manual check of the cycle. In some cycles, point I (incisura) drops below the X axis (line B in Figure 7), this case is rare and requires more studies. If the signal is in a certain cycle of the S-Hyper type, the proposed method does not specify its type within the main types of the vascular tone; other studies noted that this type can be associated with vascular diseases in the lower extremities. Moreover, this type of signal needs a specialized study to determine its characteristics and instances.

For the recorded signals of subject 1, it is clear (see Figure 8), that the vascular tone in the channel RLD is completely stable throughout the recording period. Therefore, there are no changes in the vascular tone type during the entire recording period. This rule is applicable to the rest of the recordings of the other volunteers. For subject 1, the diastolic wave in the upper part of the leg (RLU) begins to increase, and the type of vascular tone changes between hypertonic and normotonic during the recording period. For the upper extremities, the type of the vascular tone is less stable. It can be observed that the diastolic wave of this subject in most cases increases during the exhalation phase. This contributes to changing the type of the vascular tone from hypertonic to normotonic or from normtonic to hypotonic.

### 4.3. Future Work

Future studies should focus on gathering the bioimpedance data from healthy volunteers in different positions (sitting, standing, before and after exercise). Also, data from patients with specific diseases that affect the blood flow and the elasticity of blood vessels in the extremities should be acquired.

Also, the effect of the breathing phase on the ratio between the systolic wave peak and the diastolic wave peak in every cycle should be evaluated. There is research in progress that is studying the relationship between pulse wave velocity and vascular tone type.

### 4.4. Limitations

In this research, signals have been recorded only on healthy subjects who have the ability to remain in a state of rest and relaxation for the entire duration of the recording period. In fact, the proposed method requires a high relaxation level of the volunteers (meaning that the volunteer must not activate any of the limb muscles during the period of recording the signal, which extends for several minutes, as the tension of any muscle changes the signal of bioimpedance completely in the studied segment). The used device needs to be developed so that the signal can be wirelessly transmitted without the need to use connecting cables between the device and the electrodes. The reason is that any movement of the patient during recording affects stability of the recorded signal. However, this remains technically difficult because of the large number of channels used.

## 5. Conclusions

In this work, an approach has been presented to determine the vascular tone type and its temporal and spatial changes in all segments of human limbs. The study is based on the bio-impedance signals recorded using the multi-channel system Rio-32.

The work elaborates on the steps of processing the acquired signal. It shows the methodology of detecting and dividing type of the vascular tone. It also identifies the main types of signals that can be found in the extremities and the places of appearance. In order to show the results in a simple way for non-specialists in signal processing, a graphical interface has been created. This interface displays the type of the vascular tone in each limb segment. It also identifies the breathing phase during the entire recording period of the subject. The effect of the breathing phase on the vascular type in the studied case (subject 1) has been discussed. Main limitations and difficulties of this research have been outlined.

Future work is aimed at recording the signals of healthy volunteers in different conditions, and recording signals of patients with cardiovascular diseases. Finally, it also aims to determine changes that accompany the vascular tone of the limbs in healthy and pathological conditions.

## Figures and Tables

**Table 1:** Information about the recorded signals. Index of the signal (ID), its abbreviation(Abbr), segment of the body from which the signal was recorded (Segment) and main arteries in this segment (Arteries).

ID	Abbr	Segment	Arteries
1	-	Transthoracic	-
2	-	Thoracic	-
3	LAU	Upper part of Left Arm	Left Brachial artery
4	RAU	Upper part of Right Arm	Right brachial artery
5	LLU	Left Thigh	Left femoral artery
6	RLU	Right Thigh	Right femoral artery
7	LAD	Lower part of Left Arm	Left radial and ulnar arteries
8	RAD	Lower part of Right Arm	Right radial and ulnar arteries
9	LLD	Lower part of the Left Leg	Left anterior tibial and Posterior tibial
10	RLD	Lower part of the Right Leg	Right anterior tibial and Posterior tibial
11	LA	Left Arm	All arteries of left arm
12	RA	Right Arm	All arteries of right arm
13	LL	Left Leg	All arteries of left leg
14	RL	Right Leg	All arteries of right leg

## Data Availability

The full data for all subjects in this study are available on request from the corresponding author. Data of subject 1 is available in: https://www.bmt2-bmstu.ru/ahmad (accessed on 1 November 2021).

## Supplementary Materials

The following are available at https://www.mdpi.com/article/10.3390/s22010138/s1.
